# Mast cell stabilizer ketotifen fumarate reverses inflammatory but not neuropathic-induced mechanical pain in mice

**DOI:** 10.1097/PR9.0000000000000902

**Published:** 2021-06-03

**Authors:** Carolina B. Meloto, Pablo Ingelmo, Eduardo Vega Perez, Rebecca Pitt, Víctor Hugo González Cárdenas, Nada Mohamed, Susana G. Sotocinal, Valerie Bourassa, Lucas Vasconcelos Lima, Alfredo Ribeiro-da-Silva, Jeffrey S. Mogil, Luda Diatchenko

**Affiliations:** a Faculty of Dentistry, McGill University, 2001 McGill College Avenue, Montreal, QC, H3A 1G1, Canada; b Alan Edwards Centre for Research on Pain, McGill University, Montreal, QC, Canada; c Edwards Family Interdisciplinary Complex Pain Center, Montreal Children's Hospital, Montreal, QC, Canada; d Department of Anesthesia, School of Medicine, Pontifical Catholic University of Chile, Santiago, Chile.; e Fundacion Universitaria de Ciencias de la Salud, Bogotá, Colombia, Fundación Hospital Infantil Universitario de San José, Bogotá, Colombia; f Department of Psychology, McGill University, Montreal, QC, Canada; g Department of Pharmacology & Therapeutics, Faculty of Medicine and Health Sciences, McGill University, Montreal, QC, Canada; h Department of Anesthesia, Faculty of Medicine, Montreal, QC, Canada

**Keywords:** Mast cells, Ketotifen fumarate, Mechanical allodynia, Inflammatory pain, Chronic widespread pain

## Abstract

**Introduction:**

Mast cell (MC) activation could establish a positive feedback loop that perpetuates inflammation and maintains pain. Stabilizing MCs with ketotifen fumarate (KF) may disrupt this loop and relieve pain.

**Objective:**

We aimed to test the effect of treatment with KF in pain assays in mice and in a case series of patients with chronic widespread pain.

**Methods:**

The analgesic effect of KF was tested in CD-1 mice injected with formalin, complete Freund's adjuvant, or subjected to spared nerve injury. In addition, wild-type (C57BL/6) and MC-deficient (C57BL/6-*Kit*^W-sh/W-sh^) mice were injected with formalin or complete Freund's adjuvant and treated with KF. Patients with chronic widespread pain (n = 5; age: 13–16 years) who failed to respond to standard of care participated in a 16-week treatment trial with KF (6 mg/d). Ketotifen fumarate's therapeutic effect was evaluated using the patient global impression of change.

**Results:**

In the mouse experiments, KF produced dose- and MC-dependent analgesic effects against mechanical allodynia in the acute and chronic inflammatory pain but not neuropathic pain assays. In the patient case series, 4 patients reported that activity limitations, symptoms, emotions, and overall quality of life related to their pain condition were “better” or “a great deal better” since beginning treatment with KF. This was accompanied by improvements in pain comorbid symptoms.

**Conclusion:**

Treatment with KF is capable of reducing established inflammatory-induced mechanical nociception in an MC-dependent manner in mice, and it may be beneficial for the treatment of chronic pain conditions.

## Abstract 2

Supplemental Digital Content is Available in the Text.

Our preclinical findings indicate that ketotifen fumarate's analgesic effects are MC-dependent, and the case series report presented supports its use for the treatment of chronic pain.

## 1. Introduction

Low back pain,^32^ headache,^1^ vulvodynia,^27^ irritable bowel syndrome (IBS),^77^ temporomandibular disorders,^69^ and fibromyalgia^13^ are examples of chronic idiopathic pain conditions that co-occur with different types of allergy. Canonically recognized as effectors in IgE-mediated immediate type I hypersensitivity and in allergic responses such as asthma, mast cells (MCs) are bone-marrow–derived long-lived cells^21^ that release a multitude of mediators immediately upon activation, as well as de novo synthesized lipid mediators, cytokines, and chemokines.^49^ Of interest to pain are sensory neuropeptides such as substance P, calcitonin gene-related protein, and histamine (H1, H2). Located in the central nervous system and in close proximity to nerve endings and blood vessels, MC mediators could induce pain by acting directly on nociceptors, by stimulating the production of other mediators that produce pain, and/or by activating other cells.^26^ Conceivably, the activation of MCs could establish a positive feedback loop that contributes to maintain pain in chronic idiopathic pain conditions. Previous studies of sickle cell anemia, an inherited disorder characterized by chronic hemolytic anemia and lifelong pain, support this hypothesis.^78^ Thus, limiting MC activation could disrupt this loop and relieve pain. Although some reports on animal pain models do not support contribution of MCs in the pathophysiology of pain,^11,41,44^ the strong association between allergies and the risk of having or developing different chronic pain conditions^1,13,27,32,77^ indicates that activation of MCs may be relevant at least for a subgroup of patients.

Ketotifen fumarate (KF) is an antiallergic and antihistaminic agent that inhibits the calcium-dependent degranulation of MC and noncompetitively blocks histamine at the H1-receptor.^25^ Previous preclinical studies have shown that pretreatment with KF is capable of reversing allodynia in acute inflammatory^4,46^ and postoperative^58^ pain models, but those studies failed to investigate whether such an effect was MC-dependent.

In humans, treatment with KF decreased visceral hypersensitivity and improved intestinal function in patients with IBS.^35^ There are also case reports of symptoms improvement, including abdominal pain, after treatment with KF in eosinophilic colitis and gastroenteritis,^22,64^ and a randomized controlled trial showed beneficial effects of KF on neurofibroma-associated itching and pain.^63^ However, a recent study failed to demonstrate the effect of KF on fibromyalgia pain or overall fibromyalgia symptom severity, but encouraged future adequately powered randomized clinical trials using increased doses of KF.^3^

Chronic widespread pain (CWP) is an idiopathic condition whose pathogenesis has been largely related to alterations in the central nervous system, but the involvement of immune cells and the role of systemic inflammation in this condition are becoming increasingly apparent.^72^ Because both inflammatory^33^ and neuropathic^2,48^ components seem to contribute to pathophysiology of CWP, we first tested in this study the effect of treatment with KF on MCs in a series of preclinical pain assays in mice. The analgesic effect of KF is then further supported by the report of a series of cases of adolescents with CWP who were treated with KF and achieved substantial symptom improvements.

## 2. Methods

### 2.1. Preclinical study

All experiments adhered to guidelines of the Canadian Council on Animal Care (CCAC) and were approved by the Downtown Animal Use Committee at the McGill University. Adult (8–14-week old) mice of both sexes were used in all experiments. Outbred CD-1 mice were bred in-house from breeders obtained from Charles River Laboratories. C57BL/6J and MC-deficient mice (C57BL/6-*Kit*^W-sh/W-sh^) mice were purchased from The Jackson Laboratory (Bar Harbor, ME). Mice were housed with their same-sex littermates (4 animals per cage) in standard shoebox cages, maintained in a temperature-controlled (20°C ± 1°C) environment (14:10-hour light/dark cycle), and fed (Harlan Teklad 8604) and watered ad libitum. Mice were assigned to experimental conditions in a randomized fashion within the cage, and investigators were blinded to treatment (KF or vehicle).

#### 2.1.1. Drug injection

Ketotifen fumarate was injected intraperitoneally (i.p.) in a volume of 10 mL/kg physiological saline. Ketotifen fumarate doses were determined in pilot experiments using a KF dose range of 1.5 to 4.5 mg/kg.

#### 2.1.2. Formalin test

After drug injection, mice were placed on a tabletop within Plexiglas cylinders (30 cm high; 30 cm diameter) and allowed to habituate for 30 minutes. Then, 20 μL of 5% formalin was injected subcutaneously into the plantar surface of the left hind paw using a 100-μL microsyringe with a 30-gauge needle. Mice were then returned to the cylinders, and left undisturbed for 60 minutes, with behaviors recorded using digital video. Videos were later coded offline, where the first 10 seconds of every minute was monitored for the presence of licking/biting (positive sample) of the left hind paw for a total of 60 observations. The “early phase” was defined as the percentage of positive samples during the first 0 to 10 minutes postinjection of formalin; the “late phase” was defined as the percentage of positive samples during the period 10 to 60 minutes postinjection. Mice were euthanized within 10 minutes of the cessation of behavioral testing. Within 2 minutes postmortem, both hind paws were severed at the ankle joint. Each hind paw was weighed on a microbalance to the nearest 0.1 mg. Edema was expressed as the difference between the hind paw weights expressed as a percentage of body weight.

#### 2.1.3. Complete Freund's adjuvant–induced mechanical allodynia

Complete Freund's adjuvant (CFA; 50%; Sigma) was injected subcutaneously in a volume of 20 μL into the left plantar hind paw using a 100-μL microsyringe with a 30-gauge needle to create a chronic inflammatory state. Mice were tested for mechanical sensitivity of both hind paws using the von Frey test as described below, before, and 3 days post-CFA injection. Saline or KF was injected i.p. immediately after the 3-day post-CFA test, and postdrug injection measurements were taken 30 minutes later.

The up-down method of Dixon^10^ was used to measure mechanical sensitivity. Mice were placed on a perforated metal floor (with 5-mm diameter holes placed 7 mm apart) within small Plexiglas cubicles as described above, and a set of 8 calibrated von Frey (VF) fibers (Stoelting Touch Test Sensory Evaluator Kit #2 to #9; ranging from ≈0.015 g to ≈1.3 g of force) were applied to the plantar surface of the hind paw until the fibers bowed, and then held for 3 seconds. The VF threshold force required to elicit withdrawal of the paw (median 50% withdrawal) was determined twice at each time point.

#### 2.1.4. Spared nerve injury–induced mechanical allodynia

After testing for baseline mechanical sensitivity on 2 separate occasions, mice were subjected to a unilateral spared nerve injury surgery (Decosterd and Woolf, 2000) as adapted for mice (Shields et al., 2003). We spared the sural territory, so von Frey fibers (see above) were aimed at the lateral aspect of the hind paw. Mice were retested for mechanical allodynia on postoperative day 3 (the earliest time point featuring maximal allodynia) and then at several time points after drug injection.

#### 2.1.5. Rotarod test

Drug effects on motor coordination were tested using an accelerating rotarod treadmill (Acceler Rota-Rod 7650, UgoBasile) for mice.^18^ Mice were placed on the rotarod, which accelerated from 4 to 40 rpm over a period of 5 minutes, and the time spent on the rotating drum was recorded for each mouse. Performance was indicated by the latency to fall from the rotarod at 30, 60, and 90 minutes after KF injection.

#### 2.1.6. Histology

Twelve animals (6 females and 6 males) were injected with CFA (n = 8) or vehicle (SHAM; n = 4). Three days later, glabrous skin from the site of the intraplantar injection was extracted from SHAM and a subset of CFA-injected animals (n = 4). On day 3, the remainder subset of CFA-injected animals (n = 4) were treated with KF i.p. and glabrous skin from the site of the CFA injection extracted after 30 minutes. Tissues collected were postfixed in 4% formaldehyde (obtained from paraformaldehyde) in 0.1 M phosphate buffer, pH 7.4, for 24 hours. Subsequently, skin was stored in 30% sucrose in PBS, frozen, surrounded by O.C.T. compound (Tissue-Tek, Torrance, CA), and cut in 16-µm sections using a cryostat (Leica). Sections were attached to gelatin-subbed microscope slides.

To obtain a metachromatic staining of MCs, slides were soaked in a beaker of tap water for 15 seconds, and then dipped 10 times in water. Slides were then soaked for 2 minutes in 0.1% toluidine blue (Fisher BP10710) in 1% NaCl (pH 2.3). Next, slides were dipped 10 times in each of the following solutions in this specific order: distilled water, another beaker of fresh distilled water, 70% ethanol, 95% ethanol, 100% ethanol, another beaker of fresh 100% ethanol, xylene, and another beaker of fresh xylene. Slides were coverslipped with Entellan (EMS 14802) mounting medium.

#### 2.1.7. Microscopy and quantification

Bright-field micrographs were obtained using a Zeiss Axioplan 2 imaging microscope with a 63x oil-immersion objective, a high-resolution color camera, and the Zeiss Zen software version 2.3. Quantification involved 12 animals and 3 sections per animal. Two images were obtained per section, for a total of 72 images. The total number of MCs per unit area was determined from the images. Results were averaged for each animal group.

#### 2.1.8. Statistical analysis

All behavioral experiments involved the evaluation of the effects of injuries and treatment on pain behaviors and were analyzed with repeated-measures analyses of variance as appropriate (Systat v. 13). *Post hoc* comparisons were made using Systat's *post hoc* test for repeated measures with Sidak correction. Mast cell degranulation was analyzed using a two-way analysis of variance and Bonferroni's correction. An α criterion of 0.05 was adopted in all experiments.

### 2.2. Case series

#### 2.2.1. Patient characteristics

Four female and one male adolescent (13–16 years old) with CWP and additional pain conditions (ie, IBS, headaches, menstrual pain, or myofascial pain) presenting for treatment at the Chronic Pain Service of the Montreal Children's Hospital were invited for a treatment trial with incremental doses of KF. Chronic widespread pain was defined as diffuse musculoskeletal pain in at least 4 of 5 body regions and in at least 3 or more body quadrants (ie, upper–lower/left–right side of the body) and axial skeleton (neck, back, chest, and abdomen).^52^ All patients had diffuse body pain with a mean duration of 3.5 years (range: 0.5–9), overall body pain average score of 9 out of 10 on the numerical rating scale, and reported having spontaneous pain crises and sustained pain after physical activity. Additional patients' characteristics can be found in the Supplementary Material (available at http://links.lww.com/PR9/A106) and are summarized in Table 1.

**Table 1:** Clinical characteristics and self-reported pain intensity and pain-related impact before the treatment with KF.

Patient	Age	Comorbidities	Pain duration (y)	Pain intensity (NRS)	Sleep disorder	Physical disability due to pain	Academic impairment	Admission due to pain	Mood disorder	Suicidal due to pain
1	15	Allergies and cutaneous rush	2	9/10	Yes	Yes	Yes	Yes	Yes	Yes
2	16	Crohn disease	5	10/10	Yes	Yes	No	No	No	No
3	16		1	9/10	Yes	Yes	Yes	Yes	Yes	Yes
4	15		0,5	10/10	Yes	Yes	Yes	No	Yes	No
5	13	Ehrler Danlos, allergies, and cutaneous rush	9	8/10	Yes	Yes	Yes	No	Yes	No

NRS: numerical rating scale (0–10).

#### 2.2.2. Eligibility criteria

All patients were following a stable multidisciplinary treatment plan (including different combinations of pharmacological treatment, supportive psychological therapy, physical therapy, and nutritional counseling) for at least 8 weeks without reporting significant symptoms improvements before being invited to attempt treatment with KF. This includes stable doses of their medications for at least 8 weeks. Laboratory tests assessing pregnancy, blood cell count, and liver enzymes were performed, if not recently available (ie, within 6 months) in the patient's charts.

#### 2.2.3. Ketotifen fumarate treatment

Eligible patients were invited to join a 16-week open-label treatment trial with KF. The consent process was verbal and annotated in the patients' charts. Briefly, our team (P.I. and E.V.P. or V.H.G.C.) explained to patients and their parents or guardians (1) that they were being invited to attempt treatment with KF because their individualized treatments plan did not provide meaningful symptoms improvement, (2) the scientific rationale for attempting treatment with KF, a medication typically used for the treatment of asthma,^71^ and (3) all potential side effects of treatment with KF (of which the most common are drowsiness and weight gain^71^). Our team also explained that the patient would continue with their individualized treatment plan during the 16-week KF treatment trial and that no new treatments would be initiated during this period. Importantly, our team also explained that attempting this treatment was an entirely voluntary decision and deciding not to try it or to drop the treatment at any point would have no consequences to their clinical treatment. The effective oral dose of KF for the treatment of adolescents with CWP has not been established. Thus, the starting dose (1 mg/d) was chosen based on the standard treatment dose for adolescents with asthma. The safety profile of 1 mg/d KF has been largely evaluated over the years.^71^ The maximum therapeutic dose (6 mg/d) was defined based on previous studies in patients with neurofibromatosis, IBS, and eosinophilic gastroenteritis.^3,7,20,35,63,64^ Because KF may cause drowsiness, treatment started at 0.5 mg BID (1 mg/d) in the first week of treatment, 1 mg BID (2 mg/d) during the second week, 2 mg BID (4 mg/d) in the third week, and 3 mg BID (6 mg/d) in the fourth week and thereafter until completing 16 weeks of treatment. All patients were contacted every 4 weeks to monitor KF's therapeutic effect, effect on comorbid biopsychosocial symptoms, and potential adverse effects.

Therapeutic effect was evaluated using the patient global impression of change (PGIC) after 16 weeks of treatment with KF.^47^ The PGIC asks: “Since beginning treatment, how would you describe the change (if any) in ACTIVITY LIMITATIONS, SYMPTOMS, EMOTIONS and OVERALL QUALITY OF LIFE related to your painful condition?” Answer options are: 1: No change (or condition has got worse); 2: Almost the same, hardly any change at all; 3: A little better, but not a noticeable change at all; 4: Somewhat better, but the change has not made any real difference; 5: Moderately better, and a slight but noticeable change; 6: Better, and a definite improvement that has made a real and worthwhile difference; or, 7: A great deal better, and a considerable improvement that has made all the difference. Evidence of therapeutic effect was defined as options 6 or 7. In addition to KF's therapeutic effect, the patients' report of overall bodily pain intensity, frequency of pain crises, and number and dose of other medications taken during the treatment trial were also annotated.

Comorbid biopsychosocial symptoms were monitored by asking patients to rate their global improvements in pain, mood, sleep, and physical function in a scale ranging from 0% to 100%, where 0% means no change and 100% means completely normal.

## 3. Results

### 3.1. Effect of ketotifen fumarate treatment on different preclinical pain assays

We first screened the analgesic effect of treatment with KF in a series of preclinical pain assays in mice. No drug–sex interactions were noted in any preclinical assay; so in all cases, results presented below are on data combined by sex.

**Figure 1.**
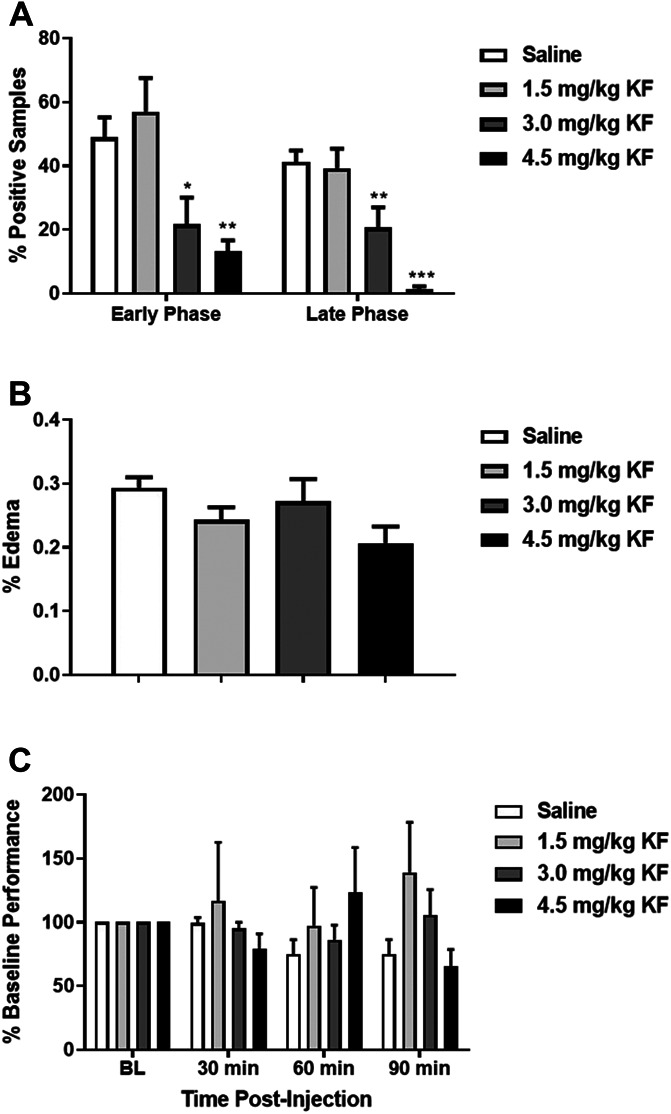
Treatment of CD-1 mice with KF reduces nocifensive behavior in a dose-dependent manner in the formalin test. Graph displays both early phase (0–10 min post‐injection) and late phase (10–60 min post‐injection) nocifensive behavior, respectively, after 5% formalin injection into the plantar hind paw. Bars represent mean ± SEM percentage of samples featuring licking behavior (% positive samples); *n* = 6 to 10 mice/dose. *P < 0.05, **P < 0.01, ***P < 0.001 compared to saline group. KF, ketotifen fumarate.

CD-1 mice were injected with KF (1.5, 3, or 4.5 mg/kg) or saline followed by formalin. Nocifensive behavior decreased in a KF dose-dependent manner both at the early (F_3,28_ = 5.8, *P* = 0.003) and late phases (F_3,28_ = 11.9, *P* < 0.001), with the highest dose resulting in significant reduction both at the early (*P* = 0.02) and late phases (*P* < 0.001) (Fig. 1A). The effect of pretreatment with KF on paw edema was not significant (F_3,28_ = 2.6, *P* = 0.07, Fig. 1B) nor did it affect rotarod performance (F_3,76_ = 1.4, *P* = 0.26, Fig. 1C). The highest dose (4.5 mg/kg) was used for all subsequent experiments.

**Figure 2.**
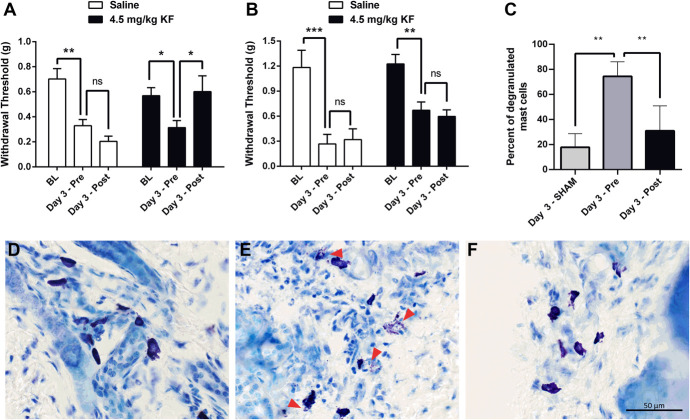
Treatment with KF reverses CFA-induced mechanical allodynia in CD-1 mice. Mechanical allodynia produced by CFA (A) but not SNI (B) is reversed by treatment with 4.5 mg/kg KF but not by saline (n = 10 mice/drug). Bars represent mean ± SEM ipsilateral hind paw withdrawal threshold at baseline (BL), 3 days after CFA (pre-injection; Pre), and 30 minutes post-injection of KF (Post). (C) Degranulation of MCs in CFA-treated animals is prevented by treatment with 4.5 mg/kg KF (n = 4). Bars represent means ± SEM percent of degranulated MCs 3 days after SHAM, 3 days after CFA (pre-injection; Pre), and 30 minutes post-injection of KF (Post). (D-F) Representative images of toluidine blue–stained skin sections. Red arrows point to degranulated MCs. *P < 0.05, **P < 0.01 as indicated; CFA, complete Freund's adjuvant; KF, ketotifen fumarate; MC, mast cell; n.s., not significant; SNI, spared nerve injury.

Mice were then tested for mechanical nociception using von Frey fibers, given a long-lasting inflammatory injury (CFA), and retested 3 days later before and 30 minutes after treatment with KF or saline. Mechanical allodynia was completely reversed by treatment with KF, but not saline, as demonstrated by the return of the VF thresholds to baseline level (drug × repeated measures: F_2,36_ = 5.5, *P* = 0.008) (Fig. 2A).

For the neuropathic pain assay, mice were tested for mechanical nociception using von Frey fibers, subjected to spared nerve injury, and retested for mechanical nociception 3 days after surgery. Mechanical allodynia was not affected by treatment with 4.5 mg/kg KF (drug × repeated measures: F_2,35_ = 0.3, *P* = 0.74) (Fig. 2B).

Histological studies revealed that KF treatment significantly decreased MC degranulation in animals injected with CFA, confirming that the mechanical allodynia reversal effect of treatment with KF was MC-dependent (Fig. 2C, D).

The modality specificity of KF's analgesic effect on animals suggests that KF may be more useful for the treatment of widespread muscular pain conditions, such as CWP, rather than those with a neuropathic component, such as sciatica pain.

### 3.2. Contribution of mast cell to ketotifen fumarate's antinociceptive effect

**Figure 3.**
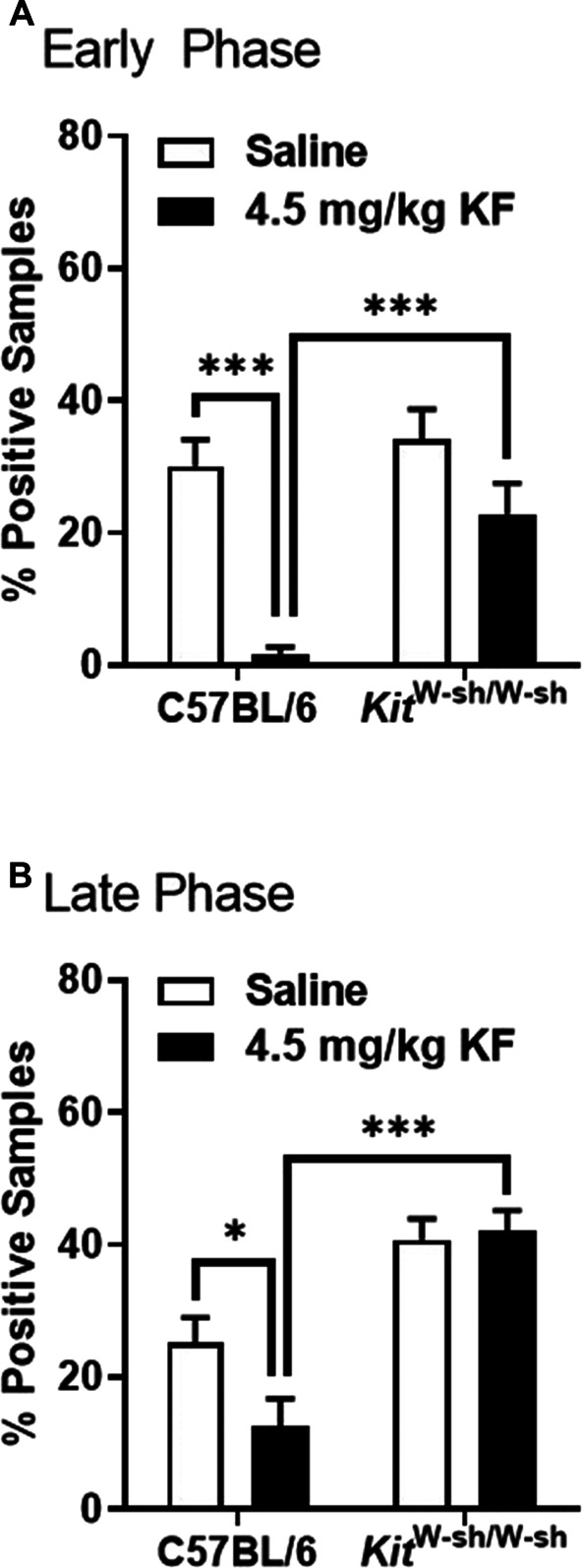
Ketotifen fumarate inhibition of formalin test responding is MC-dependent. Graphs display the early phase (A; 0–10 min post‐injection) and late phase (B; 10–60 min post‐injection) nocifensive behavior of wild-type (C57BL/6) and MC-deficient (C57BL/6-*Kit*^W-sh/W-sh^) mice after 5% formalin injection into the plantar hind paw; bars (n = 12–15 mice/genotype/drug) represent mean ± SEM percentage of samples featuring licking behavior (% positive samples). *P < 0.01, ***P < 0.001 as indicated; all other comparisons not significant. MC, mast cell.

**Figure 4.**
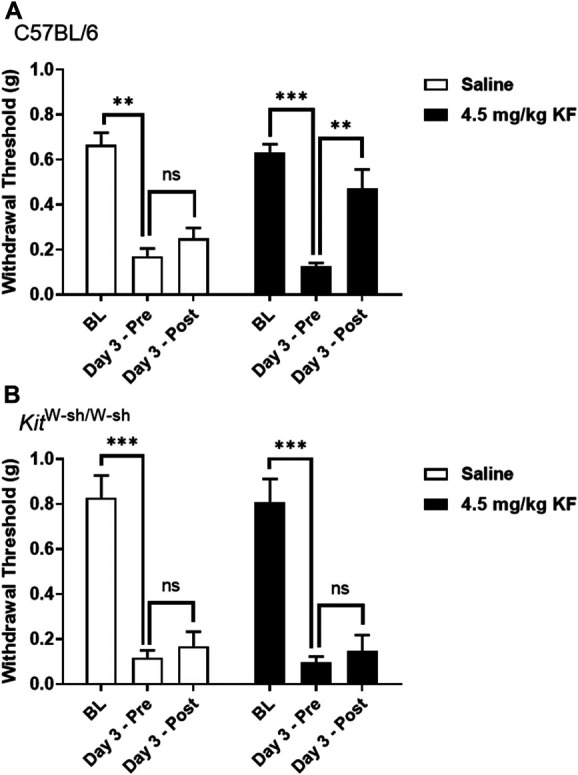
Ketotifen fumarate inhibition of CFA-induced mechanical allodynia is MC-dependent. Mechanical allodynia produced by CFA is reversed by treatment with 4.5 mg/kg KF but not saline (n = 10 mice/drug) in C57BL/6 (A; n = 12–14 mice/drug) but not in MC-deficient *Kit*^W-sh/W-sh^ mice (B; *n* = 7–8 mice/drug). Bars represent mean ± SEM ipsilateral hind paw withdrawal threshold at baseline (BL), 3 days after CFA (pre-injection; Pre), and 30 minutes post-injection of KF (Post). **P < 0.01, ***P < 0.001 as indicated; CFA, complete Freund's adjuvant; MC, mast cell; n.s., not significant; KF, ketotifen fumarate.

To further investigate whether the analgesic effect of KF on inflammatory-induced mechanical allodynia was due to its action on MC, we assessed nocifensive behavior in response to injection of formalin and CFA-induced mechanical allodynia in MC-deficient mice (C57BL/6-*Kit*^W-sh/W-sh^). In the formalin test, significant genotype × drug interactions were observed in the early phase (F_1,47_ = 4.6, *P* = 0.04) and late phase (F_1,47_ = 6.2, *P* = 0.02). In both phases, no genotype differences in formalin sensitivity were observed, but KF was found to inhibit response to formalin test in wild-type but not MC-deficient mice (Fig. 3). In the CFA mechanical allodynia test, a significant genotype × drug × repeated measures interaction was observed (F_2,72_ = 3.8, *P* = 0.03), such that KF but not saline reversed mechanical allodynia in wild-type but not MC-deficient mice (Fig. 4). These findings suggest that KF's analgesic effect on inflammatory-induced mechanical allodynia is at least partly mediated by MC.

### 3.3. Effect of ketotifen fumarate treatment in a case series of patients with chronic widespread pain

All patients were able to achieve KF's maximum therapeutic dose (6 mg/d) within 4 weeks. Ketotifen fumarate's therapeutic effect was clinically evident after 6 to 8 weeks of treatment, when patients no longer reported spontaneous pain crisis and reported initial positive changes on their mood, sleep quality, physical activity, and school attendance. At the end of 16 weeks of treatment (Table 2), 4 out of 5 patients scored either 6 or 7 on the PGIC. Three patients reported having no more pain and another 2 reported 50% reduction on their average pain intensity. Two patients reduced by at least 50% the number and doses of prior pain medications, and another 3 were completely free of other medications. Four patients quantified their global improvements in mood, sleep, and physical function in the range of 60% to 100%. All patients went back to their normal school activities, including physical activities at school, and 4 of them were able to return to their previous competitive sport activities. Notably, all patients reported being completely or almost completely free of spontaneous pain crises.

**Table 2:** Self-reported changes in PGIC (Patient Global Impression of Change), average pain reduction, need for pain medications, physical function, sleep, and mood after 16 weeks of treatment with KF.

Patient	PGIC	Pain reduction (%)	Need for pain medications	Physical disability due to pain	Change in sleep (%)	Change in mood (%)	Normal school activities
1	7	100	Yes	No	80	90	Yes
2	3	50	Yes	Minimal	60	0	Yes
3	6	100	No	No	100	90	Yes
4	7	50	No	No	80	80	Yes
5	7	100	No	No	100	100	Yes

Three patients reported drowsiness during the first 2 to 3 weeks of treatment. One patient had nausea for few days at the beginning of the treatment and one patient gained 5 kg during the treatment. There were no other adverse effects reported during treatment.

One patient is still under treatment with KF and 4 patients discontinued it 6 months after the end of the trial. Three of them were free of pain or other symptoms one year after discontinuing KF. Two patients reported the recurrence of pain and other symptoms a few months after discontinuing treatment.

This case series report is in line with our preclinical findings and encourages future clinical trials to formally assess the effect of KF for the treatment of nonneuropathic pain conditions.

### 3.4. Discussion

Inspired by the highly reported but poorly investigated comorbidity between different types of allergies and multiple chronic pain conditions,^1,13,27,32,77^ we sought out to investigate the effect of limiting the activation of MCs with KF on nociception preclinically and to support our preclinical findings with preliminary clinical data. Our findings show that pretreatment with the MC stabilizer KF reduces nocifensive behavior in acute formalin inflammatory pain assay in mice. Furthermore, this is the first report showing that KF reverses mechanical allodynia even after CFA-induced inflammation develops. Importantly, the analgesic effect of KF was specific to inflammatory pain, suggesting that treatment with KF may be better indicated for chronic pain conditions of nonneuropathic etiology. In addition, our findings support a direct role for MCs in pain, as KF inhibited the activation of MCs and its analgesic effect was evident in wild-type but not MC-deficient mice. Our findings are in line with a previous study showing that preventing MC activation also ameliorates sickle cell–induced pain.^78^ Our findings and others^41,44^ indicate that MCs do not account solely for the development of inflammatory-induced pain, as it developed in both wild-type and MC-deficient mice. Indeed, other immune cell types contribute to the development of inflammatory-induced pain.^15,40,74,81^ Nevertheless, treatment with KF reversed inflammatory-induced mechanical pain, suggesting that limiting MC activation is crucial to resolve the ongoing inflammation and consequently, the ongoing pain. Thus, the translational value of our findings is substantial as it suggests that stabilizing MCs in patients with ongoing chronic pain conditions may be useful to promote analgesia. Our hypothesized role for MCs in ongoing chronic pain was further supported by the case series of adolescent patients with CWP treated with KF who reported substantial symptom improvements. However, whether treatment with KF is truly beneficial and safe for the treatment of adolescent or adult chronic pain patient remains to be investigated in clinical studies using greater scientific rigor (ie, randomized controlled trials) and is not recommended at this time.

Our findings are consistent with previous studies reporting that pretreatment with KF reduces acute inflammatory nociception^4,46^ in rats. Prevention of upregulation of interleukin-6, tumor necrosis factor alpha, and nerve growth factor (NGF)—mediators whose role in inflammation and hyperalgesia have been well documented—^17,68^ has been suggested as one of the mechanisms whereby KF inhibits acute inflammatory pain.^46^ Pretreatment with KF also reduced acute postoperative hyperalgesia in mice,^58^ and its antihyperalgesic effect lasted longer than that of cromoglycate.

The antiallodynic effect of treatment with KF on ongoing CFA-induced inflammatory hypersensitivity had not been assessed before, but studies have shown this effect in other pain assays. Treatment with KF 4 weeks after radiation-induced injury reduced colorectal mechanical allodynia in rats.^19^ Furthermore, treating rats with visceral hypersensitivity as result of stress with KF abolished allodynia, indicating that MC may also be implicated in stress-induced pain.^53^

The exact mechanisms whereby KF reduces nociception remain to be understood, and most if not all studies aimed at elucidating them were done in the context of asthma and allergic disorders. In these conditions, KF efficacy has been associated with decreased levels of histamine, tryptase,^14,25^ tumor necrosis factor alpha,^23,54^ macrophage-derived chemokines,^31^ and interleukin-8.^56^ Most importantly, KF has MC-stabilizing properties,^25^ and a relationship between MC and various clinical pain conditions has been previously established.^5,6,8,9,16,24,27–29,60,70,75,76^ This led to many studies that demonstrated MCs' role in preclinical models of different pain conditions.^11^ Additional evidence of the role of MC in the pathophysiology of different pain conditions is provided by preclinical models of pelvic pain,^16,55,65,79^ abdominal pain,^30^ arthritis pain,^42^ and induced inflammatory pain^12^ using MC-deficient mice, in which pain responses were absent or significantly reduced in animals lacking MCs but restored upon reconstitution with bone marrow–derived cultured MCs.

The mechanisms underlying MCs' contribution to pain seem to be multileveled. Degranulation of MCs produced long-lasting excitation of meningeal nociceptors, activating central downstream pathways leading to hypersensitivity in migraine.^37,38^ Histamine release from MCs contributed to the development of symptoms of pelvic pain.^43,66^ Increased levels of histamine, serotonin, and tryptase were also implicated in postoperative pain, and stabilizing MCs prevented this increase and the resultant allodynia.^57,58^ Using a preclinical model of vulvodynia, it was also suggested that increased local nerve density may be MC-dependent, and treatment with another MC-stabilizer, cromoglycate, reduced local hypersensitivity.^45^ Although one could suggest that the mechanisms underlying MC contribution to pain may be specific to pain modality, these mechanisms are more likely to be pleiotropic and stabilizing MCs is likely to have multiple benefits for pain management.

Controversial evidence exists concerning the MCs' contribution to pain. Although the link between NGF and MC activation has been extensively implicated in different models of hyperalgesia,^36,39,59,67,80^ recent studies have shown that selective depletion of MCs does not reduce NGF-induced peripheral sensitization in vivo,^41,44^ nor is NGF capable of activating MCs, as they do not express NGF receptors.^41^ These studies also showed that CFA-induced mechanical and thermal allodynia^41^ and formalin-induced thermal allodynia^44^ were not different between selectively MC-depleted and wild-type animals, suggesting that MCs contribute little to the sensitization of peripheral nociceptors. The contribution of MCs to pain via its proteases is also debatable, as they were found not to be essential for acute pain response in the formalin model.^44^

Our preclinical findings are supported by a case series of patients with CWP treated with KF, who achieved substantial symptom improvement. This is in line with reports of cases of patients with long-lasting sickle cell anemia pain that improved after the inhibition of MC activation.^50,73^ One clinical trial of KF in adult patients with fibromyalgia has been conducted but did not find significant changes on pain sensitivity or functional status of patients receiving 4 mg/d KF for 8 weeks. The authors speculated that these results could be explained by an inadequate degree of MC stabilization, due to a short treatment time or low daily dose, or simply due to a small sample size.^3^ Another explanation is that perhaps not all symptoms presented by patients with CWP can be treated with KF. Another study has reported that 8 weeks of treatment with KF at a higher dose (6 mg/d) significantly increased the threshold for rectal discomfort in patients with IBS, decreased abdominal pain, and improved quality of life.^35^ Benefits of KF to neurofibroma-associated pain^62,63^ have also been reported. Thus, in our case series, we used a higher dose of KF and for a longer time than those used in the FM clinical trial.^3^ Our results encourage future clinical trials of KF to treat different chronic pain conditions using well-defined treatment outcome measures, and indicate that treatment should be carried on for at least 16 weeks with higher daily doses, possibly 6 mg/d as used in the case series reported here.

This study has important limitations that should be discussed. The development of lineage progenitors into mature MCs is dependent on the growth stem cell factor binding to its receptor, Kit. The mouse strain used here (*Kit*^W-sh/W-sh^) carries a mutation that leads not only to selective reduction of *Kit* expression and consequently, MC deficiency, but also to other abnormalities related to the altered *Kit* expression on progenitor populations of other immune cell lineages. Hence, the validity of this mouse strain to distinguish unequivocally the contributions of MCs to a given phenotype from the pleiotropic functions of Kit in other cell lineages is debatable.^34^ A stabilizing effect of KF on the release of mediators secreted by neutrophils^51^ and eosinophils^61^ has also been detected under some conditions, although this effect is evidently stronger in MCs. Thus, we cannot completely rule out that the analgesic effect of KF observed here might be partly mediated by its effects on other cell types. Finally, our preclinical findings are supported by a case series of CWP adolescents treated with KF who achieved clinical improvements. However, the reliability of such narrative studies is limited, and randomized clinical trials are warranted to further support KF's efficacy for the treatment of painful conditions.

In conclusion, stabilizing MC with KF before and after inflammation develops reduces acute nocifensive behavior and reverses mechanical allodynia, respectively, in a *Kit*-dependent manner. Combined with the report of a case series of patients with CWP who achieved important clinical improvements after treatment with KF, our findings support a role for MC in musculoskeletal pain and encourage future clinical studies aimed at testing KF's efficacy in reducing pain in multiple nonneuropathic chronic pain conditions. They also encourage the development of new and more efficient therapeutics that limit the activation of MC in a selective manner.

## Disclosures

The authors have no conflicts of interest to declare.

## Appendix A. Supplemental digital content

Supplemental digital content associated with this article can be found online at http://links.lww.com/PR9/A106.

## Supplementary Material

SUPPLEMENTARY MATERIAL
