# Supplementary material for: Mast cell stabilizer ketotifen fumarate reverses inflammatory but not neuropathic-induced mechanical pain in mice
Source: Pain Rep. 2021 Jun 3;6(2):e902. doi: 10.1097/PR9.0000000000000902 (PMC8177879; doi:10.1097/PR9.0000000000000902)
Supplement: SUPPLEMENTARY MATERIAL [file painreports-6-e902-s001.docx]

**Supplementary Methods**

***Case series***

Additional patients’ characteristics included moderate-to-severe physical impairment as defined by a Functional Disability Inventory score >12 points [1] and missing school days to the point that academic performance was affected in four out of five patients. All patients reported fatigue, significant sleep disturbances (problems to initiate sleep and awakening due to pain), cognitive changes and mood disorders, including suicidal ideation and attempts in two of them. In all cases, physical examination was within normal limits except for tactile hyperalgesia and allodynia in all patients, and joint hyperlaxity in one case (Manuscript Table 1).

Prior to initiating treatment with KF, all patients were following a multidisciplinary treatment plan for an average of nine months (range: 6-11). This individualized treatment plan was tailored to the reduction of symptoms and maintenance of adequate physical and social function and included different combinations of pharmacological treatment, supportive psychological therapy, physical therapy, and nutritional counseling In specific, pharmacological treatments used before and during treatment with KF, as well as after the 16-week period of KF treatment are shown in Supplementary Table 1. All patients received face-to-face cognitive behavioral therapy including education about the biopsychosocial factors involved in pain perception, expression, and management; self-relaxation, self-hypnosis, and biofeedback; and insomnia management through stimulus control, mindfulness, and acceptance and commitment therapy-based strategies. All patients were also part of an individualized and gradual exercise program specifically created by the physiotherapy team to regain function. Individual programs were integrated into activities that are of interest to the patient to optimize adherence. Two patients also received nutritional counseling and were following gluten free and lactose free diets.

**Supplementary Table 1.** Pharmacotherapy before/during and after the 16-week KF treatment.

| **Patient** | **Before/during ketotifen** | **After treatment** |
| --- | --- | --- |
| **1** | Celecoxib, Amitriptyline, Nabilone, Gabapentin, Tramadol, Quetiapine, ketamine, Duloxetine | Ketotifen |
| **2** | Amitriptyline, Paracetamol, Tramadol, Gabapentin | Amitriptyline, NSAIDs |
| **3** | Tramadol, Paracetamol, Fluoxetine, Pregabaline, Nabilone | No meds |
| **4** | Amitrityline, Tramadol, Paracetamol, Nabilone | No meds |
| **5** | Rizatriptan, Amitriptyline, Paracetamol, Nortriptyline,  Pregabaline, Tramadol | No meds |

**References**

[1] Walker LS, Greene JW. The functional disability inventory: measuring a neglected dimension of child health status. J Pediatr Psychol 1991;16(1):39-58.
